# Clinical outcomes of catheter ablation of paroxysmal atrial fibrillation in very young population compared to older population: a prospective study

**DOI:** 10.1186/s43044-019-0017-z

**Published:** 2019-09-16

**Authors:** Lamyaa Allam, Rania Samir, Ahmed Nabil Ali

**Affiliations:** Cardiology Department, Ain Shams University, 48 Mohammed Elnadi street, s6th zone, Nasr City, P0 11371 Egypt

**Keywords:** Paroxysmal atrial fibrillation, Young adults, AF recurrence, RF ablation, 3D anatomical mapping

## Abstract

**Background:**

Data on procedural characteristics and clinical outcome of catheter ablation of atrial fibrillation (AF) in adults younger than 35 years has not been sufficiently addressed. The aim is to assess procedural characteristics and clinical outcome of catheter ablation of paroxysmal atrial fibrillation in young adults in comparison to older adults.

**Results:**

Seventy-six consecutive patients with symptomatic paroxysmal AF underwent pulmonary vein isolation (PVI) at Ain Shams University Hospitals from 2013 till 2016. They were divided into the two groups, young population group (mean age 31.6 ± 4.2 years, 77% men) and older population group (mean age 49 ± 8.4 years, 74% men). Clinical data before and during the procedure were recorded. Follow-up was based on outpatient visits including 24 h Holter, ECG at 3, 6, and, 12 months post single ablation procedure. Recurrence was defined as any AF/atrial tachycardia episode > 30 s following a 3-month blanking period. Body mass index, CHA2DS2-VASc score, and left atrial volume were higher in the older population group [*P* values 0.019, < 0.001, and 0.001, respectively]. The presence of low-voltage areas was found only in 22% of the older population group and not in the younger group [*P* 0.02]. All patients were followed up for 1 year; 1-year arrhythmia-free survival after a single procedure was 83.3% (25/30) and 78.3% (36/46) in the older group [P 0.75]. No complications were recorded in both groups. Redo AF ablation were done for four patients in the old group and one patient in the young group.

**Conclusions:**

Catheter ablation of AF in very young adults is associated with higher 1-year success rates but comparable to success rates in older populations. AF ablation for PAF is effective in very young adults.

## Background

Atrial fibrillation (AF) is the most frequently encountered cardiac arrhythmia [1]. AF is strongly age-dependent affecting 4% of individuals older than 60 years and 8% of persons older than 80 years [2]. The prevalence of AF is 0.1% in persons younger than 55 years, 3.8% in persons 60 years or older, and 10% in persons 80 years or older [3]. Catheter ablation incorporating pulmonary vein isolation (PVI) is an established treatment option for symptomatic AF. Current guidelines recommend catheter ablation for drug-refractory AF in adults and as first-line therapy in selected patients with paroxysmal AF (PAF) [4]. However, data on procedural characteristics and clinical outcome of catheter ablation of AF in adults younger than 35 years has not been sufficiently addressed in previous studies except for only one study [5] which showed catheter ablation of AF is effective and associated with an acceptable complication rate in this population. But there are no studies comparing these data with that of AF in older patients.

Based on these data, this study was designed to further study procedural characteristics and clinical outcome of catheter ablation of paroxysmal AF in young adults less than 35 years of age in comparison to older adults.

## Methods

### Study population

This was a prospective single-center study which included 80 consecutive patients who underwent radiofrequency catheter ablation (RFCA) of symptomatic PAF at our Department in Ain Shams University Hospitals during the period from 2013 till 2016.

PAF was defined according to the recommendations of the 2012 HRS/EHRA/ECAS Expert Consensus Statement on Catheter and Surgical Ablation of Atrial Fibrillation [6]. Structural heart disease (SHD) was defined as having hypertensive heart disease, coronary artery disease, cardiomyopathy, and grown-up congenital heart disease as described by Hoffmann BA et al. [7].

All patients were divided into two groups according to age at the time of the index ablation procedure. Young population group included 34 patients aged < 35 years while the old group included 46 patients ≥ 35 and ≤ 70 years old.

Trans-esophageal echocardiography was performed in all patients to rule out left atrial (LA) thrombus before ablation. In patients on vitamin K antagonists, anticoagulation was stopped 3 days prior to ablation and replaced by intravenous heparin to maintain a partial thromboplastin time of 2–3 times of the normal value or bridged with low molecular weight heparins. Ablation was performed under therapeutic INR values of ≤ 2. The study was performed under general anesthesia after obtaining informed written consent. All patients consented for the procedure and the study protocol.

### Mapping

First, we evaluated patients for the presence of supraventricular tachycardia (SVT) and inducibility of AVNRT/AVRT in young patient’s cohort. In case of AVNRT/AVRT, slow pathway ablation or ablation of the accessory pathway (AP) was performed and those patients were excluded from the study.

Trans-septal access to the left atrium was obtained with standard techniques followed by the introduction of the long vascular sheath (PREFACE® Sheaths, Biosense Webster, USA).

Repetitive intravenous heparin was administered to maintain an activated clotting time of 300 to 350 s. Mapping and catheter ablation of AF was performed using 3D electro-anatomical (3D EA) mapping system (CARTO^R^ 3 System, Biosense Webster, Diamond Bar, CA, USA)

Simultaneous surface ECG and bipolar intracardiac electrogram (EGM) recordings (filtered between 30 and 500 Hz) were amplified and displayed using the Prucka CardioLab System (GE Medical Systems, Milwaukee, WI, USA).

Mapping was performed during sinus rhythm using a 3.5-mm-tip catheter (ThermoCool Navi-Star, Biosense Webster Inc., Diamond Bar, CA, USA) to create a voltage map. In each case, we tried to obtain an evenly distributed map throughout the left atrium.

Voltage was measured from the peak-to-peak bipolar signal filtered at 30–400 MHz with the ablation catheter using an interpolation threshold of 10 mm. Bipolar voltage amplitudes > 0.5 mV were considered “normal,” whereas amplitudes ≤ 0.5 mV were defined as “diseased” (0.1–0.5 mV) or “scar” (< 0.1 mV). A low-voltage area (LVA) was defined by ≥ 3 adjacent mapping points that each had a voltage of ≤ 0.5 mV. In order to characterize regional voltage distribution, the LA shell was divided into seven distinct anatomic segments: septum, anterior wall, floor, lateral wall, posterior wall, roof, and peri-mitral annular (MA) region [8]. Activated clotting time (ACT) measurement was performed every 20–30 min, and heparin dose was adjusted to the desired target ACT.

After the 3D reconstruction of the LA, each pulmonary vein (PV) ostium was tagged on the map.

Lasso catheter was positioned across the ostium of each PV aiming at recording pulmonary vein potentials (PVPs).

### RF ablation protocol

RF ablation approach chiefly involved pulmonary vein isolation (PVI) in all patients plus individually tailored substrate modification guided by endocardial voltage mapping in the event of encountering identifiable scars or low-voltage areas.

PVI was performed first at the antrum of left PVs followed by the right-sided PVs using RF energy of 25–30 W with an external irrigation flow rate of 15–17 ml/min. RF current was applied continuously with repositioning of the catheter tip every 30–60 s. Special caution was done at the posterior wall near the esophagus as power was reduced to 25 W with continuous repositioning of the catheter tip every 20 s. Disappearance or dissociation of PV potentials on Lasso catheter was defined as acute PVI success. Then, in case of detection of low-voltage areas, additional substrate modification was performed by placement of strategic linear lesions connecting non-excitable tissue or electrical isolation of the LVA depending on the location, the shape, and the extent of an LVA. In LVAs located in the posterior wall, substrate modification was achieved by creating box lesions including the LVAs electrically isolating the posterior wall and verification of entrance or exit block. Other LVA ablation strategies depended on the size of the scar; if the LVA was small, ablation aimed at encircling the LVA with ablation lesions aiming at electrically isolating the area, and if the LVA was large, isolation from the remainder myocardium was achieved by linear ablation lesions that were connected to non-conducting atrial structures. Then, conduction block across linear lesions (entrance and exit block) and electrical isolation of circumscribed areas were confirmed.

After ablation, patients who had AF during the study underwent electrical cardioversion (CV) in order to verify PVI in SR, and PVI was reassessed in all patients after a 30-min waiting period. Confirmation of PVI was defined by the absence of PVPs on the Lasso catheter during pacing from a coronary sinus or an LA catheter (entrance block) and failure of capturing the atrium through pacing from all poles of Lasso catheter inside each PV (exit block).

Post AF ablation, all patients were followed up every 3 months for a period of 1 year as regards AF recurrence. The primary endpoint was freedom from AF recurrence during a 12-month follow-up period after a single ablation procedure. Arrhythmia recurrence was defined as any episode of AF > 30 s documented beyond a 90-day post ablation blanking period (BP) on 12 lead surface ECG or 24-h Holter recording that was obtained every 3 months during the period of follow-up.

### Statistical analysis

All data were revised and statistically analyzed using IBM SPSS software package version 20. Continuous data are expressed as mean and standard deviation. Population proportions were presented as a percentage. Comparisons between groups for categorical variables were assessed using chi-square test (Fisher or Monte Carlo). Mann-Whitney test was used to compare two groups for abnormally distributed quantitative variables. Student *t* test was used to compare two groups for normally distributed quantitative variables. Significance of the obtained results was judged at the 5% level. *P* value < 0.05 was considered significant, and *P* value < 0.01 was considered highly significant.

## Results

### Patient characteristics

Patient characteristics are depicted in Table 1.

**Table 1:** Baseline clinical characteristics of the study groups

Characteristic	Young group	Old group	*P* value
Age at ablation time (years)	31.6 ± 4.2 (20–35)	49 ± 8.4 (36–66)	< 0.001
Gender			0.85
Male	23 (76.6 %)	34 (73.9%)	
Female	7 (23.3%)	12 (26.1%)	
BMI (kg/m^2^)	27 ± 3.7	29.2 ± 4.1	0.019
BMI > 25 kg/m^2^	10 (33.3%)	18 (39.1%)	
BMI > 30 kg/m^2^	8 (26.6%)	21 (45.7%)	
Arterial hypertension	15 (50%)	28 (60.9%)	0.43
Diabetes mellitus	5 (16.6%)	10 (21.7%)	0.63
Smoking	5 (16.6%)	14 (30.4%)	0.201
Thyroid disease	0	0	
Structural heart disease	1 (3.3%)	5 (10.8%)	0.154
IHD	0	4	
DCM	0	0	
HCM	1	1	
VHD	0	0	
AF			
Duration (months)	36 (12–120)	36 (6–120)	0.3
Frequency/month	4 (2–6)	3 (1–6)	0.34
EHRA score	3 (27 patients)	3 (38 patients)	0.29
LA volume (ml)	39.6 ± 3.1	52.8 ± 4.9	0.001
LVEF%	65.6 ± 3.2	64.2 ± 4.5	0.138
CHA2DS2-VASc score	0.93 ± 0.75	2.11 ± 1.32	< 0.001
	Median 1	Median 2	
Beta blockers	2 (6.7%)	4 (8.7%)	
Class I AAD	16 (53.3%)	20 (43.5%)	
Class III AAD	12 (40%)	26 (56.5%)	

*BMI* body mass index, *IHD* ischemic heart disease, *DCM* dilated cardiomyopathy, *HCM* hypertrophic cardiomyopathy, *VHD* valvular heart disease, *AF* atrial fibrillation, *LA* left atrium, *LVEF* LV ejection fraction, *AAD* antiarrhythmic drugs

The mean age at the time of the index procedure was 31.6 ± 4.2 years in young population group while it was 49 ± 8.4 years in the older population group with *P* value < 0.001.

Young population group included 34 patients with PAF, but four patients of young populations were excluded from the study as three patients had AVRT and one patient had AVNRT diagnosed during EP study, while the rest of patients underwent RF ablation of AF. Old population group included 46 patients.

Young population group included 23 (76.6 %) males and 7 (23.3%) females, and old population group included 34 (73.9%) males and 12 (26.1%) females with no significant difference between both groups regarding gender distribution (*P* value 0.85).

Body mass index (BMI) was significantly higher in the old population group (29.2 ± 4.1 kg/m^2^) than in young population group (27 ± 3.7 kg/m^2^) with *P* value 0.019. In the young population group, 12 patients (40%) had normal BMI and ten (33.3%) patients were overweight while eight (26.6%) patients were obese [BMI ≥ 30 kg/m^2^]. In the old population group, seven patients (15.2 %) had normal BMI and 18 (39.1%) patients were overweight while 21 (45.7%) patients were obese.

CHA2DS2-VASc score [9] was significantly higher in old population group (2.11 ± 1.32, median 2) as compared to young population group (0.93 ± 0.75, median 1) with *P* value < 0.001.

### Pre-procedural echocardiographic parameters (Table 1)

Left atrial volume was significantly higher in the old population group (52.8 ± 4.9 ml) than in the young population group (39.6 ± 3.1 ml) with *P* value 0.001, while measured left ventricular systolic function (EF) was 65.6 ± 3.2 % in the young population group and 64.2 ± 4.5 % in the old population group with *P* value 0.138.

### Mapping and ablation outcomes (Table 2)

Mapping and ablation were guided by 3D EA mapping using CARTO 3 System in all study populations. Complete PVI was achieved in all procedures of both groups (old population group 46/46, 100% and young population group 30/30, 100%). Entrance block and exit block were achieved in all study population of both groups. Demonstration of dissociated PVPs was seen in eight patients in the young population group (26.6%) and 33 patients in the old population group (71.7%).

**Table 2:** Procedural data of AF ablation in both study groups

Procedural data	Young group	Old group	*P* value
Fluroscopy time (minutes)	49.5 ± 28.7	49.8 ± 30.6	0.98
Procedural time (minutes)	159.9 ± 33.1	170.9 ± 30.1	0.152
PV anatomy			0.8
Four separate PV ostia	23 (76.7%)	37 (80.4%)	
Common LPV ostium	6 (20%)	8 (17.4%)	
Common RPV ostium	1 (3.3%)	1 (2.2%)	
Voltage map data			0.02
Presence of LA scar	0	10 (21.7%)	
Total scar size (% of total atrial mass)		10.2 (5–20) %	
PVI attempted	30 (100%)	46 (100%)	
PVPs			0.946
Eliminated	22 (73.3%)	33 (71.7%)	
Dissociated	8 (26.6%)	13 (28.3%)	
Exit block	30 (100%)	46 (100%)	

*PV* pulmonary veins, *LPV* left pulmonary veins, *RPV* right pulmonary veins, *LA* left atrium, *PVI* pulmonary vein isolation, *PVPs* pulmonary vein potentials

In young population group, anatomical mapping revealed four separate PVs in 23 out of 30 patients (76.7%), common left PV ostium in six patients (20%), and common right PV ostium in one patient (3.3%). While in the old population group, anatomical mapping revealed four separate PVs in 37 out of 46 patients (80.4%), common left PV ostium in eight patients (17.4%), and common right PV ostium in one patient (2.2%) (*P* value 0.8).

**Fig. 1:**
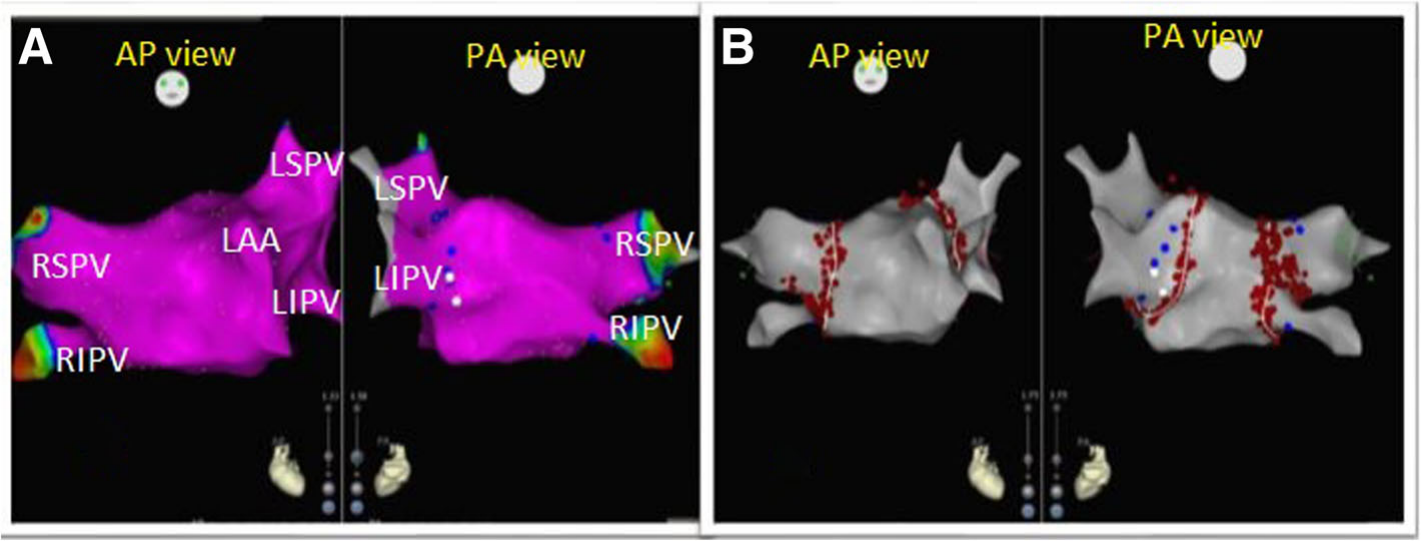
Three-dimensional mapping of the left atrium (LA) and pulmonary veins (PVs) in a patient from the young population group with paroxysmal AF. **a** Normal bipolar voltage map of LA and four separate PVs. **b** Circumferential pulmonary vein isolation was performed in the same patient. LSPV: left superior pulmonary vein, LIPV: left inferior pulmonary vein, RSPV: right superior pulmonary vein, RIPV: right inferior pulmonary vein, LAA: left atrial appendage, AP view: anteroposterior view, PA view: posteroanterior view

**Fig. 2:**
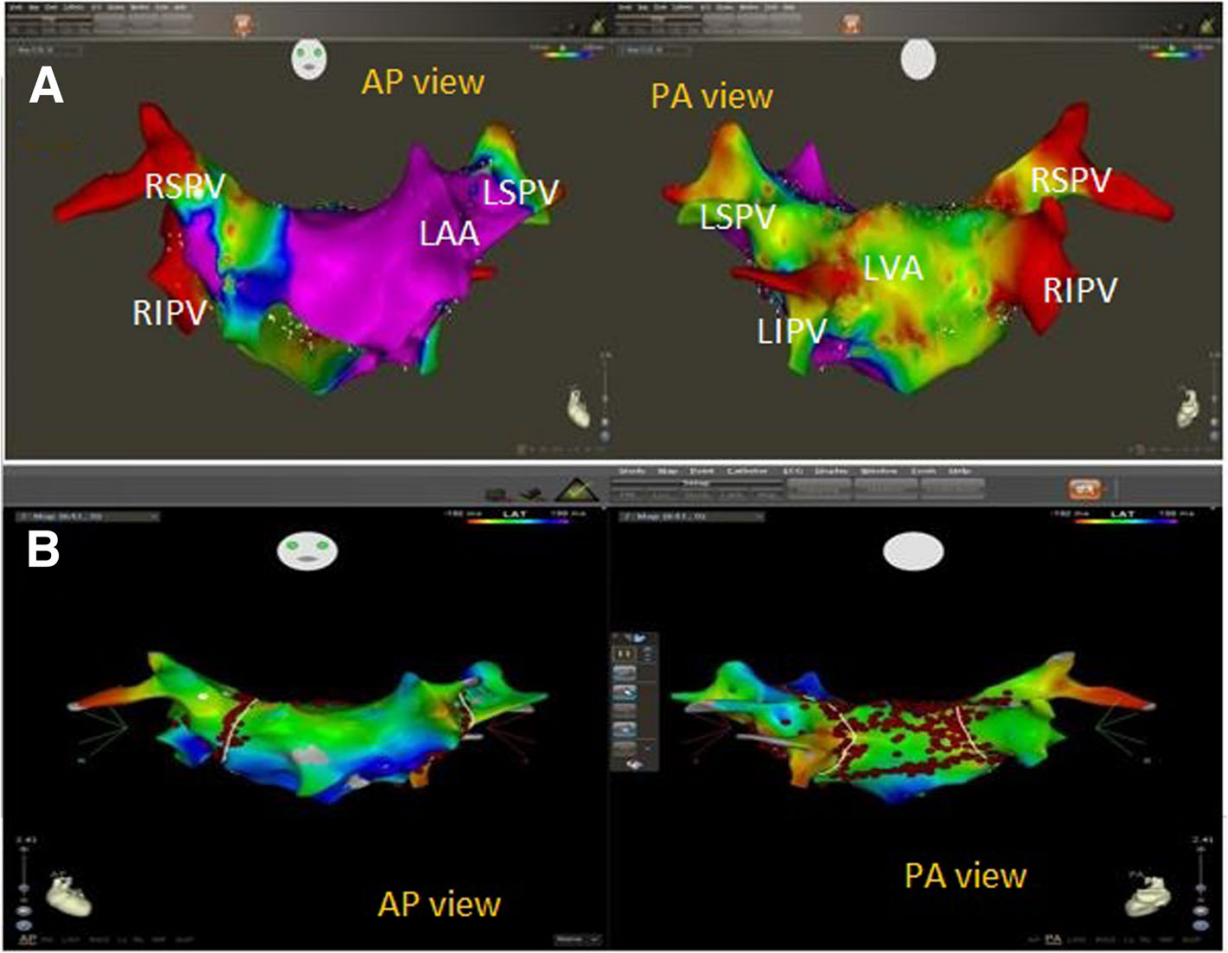
Three-dimensional mapping of the left atrium (LA) and pulmonary veins (PVs) in a patient from the older population group with paroxysmal AF. **a** Abnormal bipolar voltage map of LA showed a large low-voltage area (LVA) in the posterior wall. **b** Combined circumferential pulmonary vein isolation and electrical isolation of this LVA (box isolation) were performed in the same patient. LSPV: left superior pulmonary vein, LIPV: left inferior pulmonary vein, RSPV: right superior pulmonary vein, RIPV: right inferior pulmonary vein, LAA: left atrial appendage, LVA: low-voltage area, AP view: anteroposterior view, PA view: posteroanterior view

As regards voltage map of LA, it was normal in all patients of the young population group, while in the old population group, 10 out of 46 patients (21.7%) had a scar with *P* value 0.02. Average total LA scar size was 10.2 (5–20%) of total LA mass. Low-voltage areas were predominantly located at the LA posterior wall (four patients), roof (two patients), interatrial septum (three patients), and anterior wall near MV annulus (one patient). Four patients had small scars and ablation lesions electrically isolated the area, while two patients had large scars which were isolated from the remainder myocardium by linear ablation lesions that were connected to non-conducting atrial structures (Figs. 1 and 2).

Total fluoroscopy time was 49.5 ± 28.7 min in young population group and 49.8 ± 30.6 min in the old population group with *P* value 0.98, and total procedural time was 159.9 ± 33 min in young population group while it was 170.9 ± 30 min in old population group (*P* value 0.15).

### Follow-up (Table 3)

After a follow-up duration of 1 year following the ablation procedure, 25 of 30 patients in the young population group were in stable sinus rhythm with a 1-year arrhythmia-free survival after a single procedure of 83.3%, and 36 of 46 patients (78.3%) in old population group were in stable SR without AADs with 1-year arrhythmia-free survival of 78.3% (*P* value 0.75.).

**Table 3:** Atrial fibrillation (AF) recurrence over 1 year among both study groups

AF recurrence	Young population group	Old population group	*P* value
AF recurrence	5 (16.6%)	10 (21.7%)	0.75
Response to AADs	4 (80%)	6 (60%)	0.53
Need for redo AF ablation	1 (3.3%)	4 (40 %)	0.95

*AF* atrial fibrillation, *AADs* antiarrhythmic drugs

**Fig. 3:**
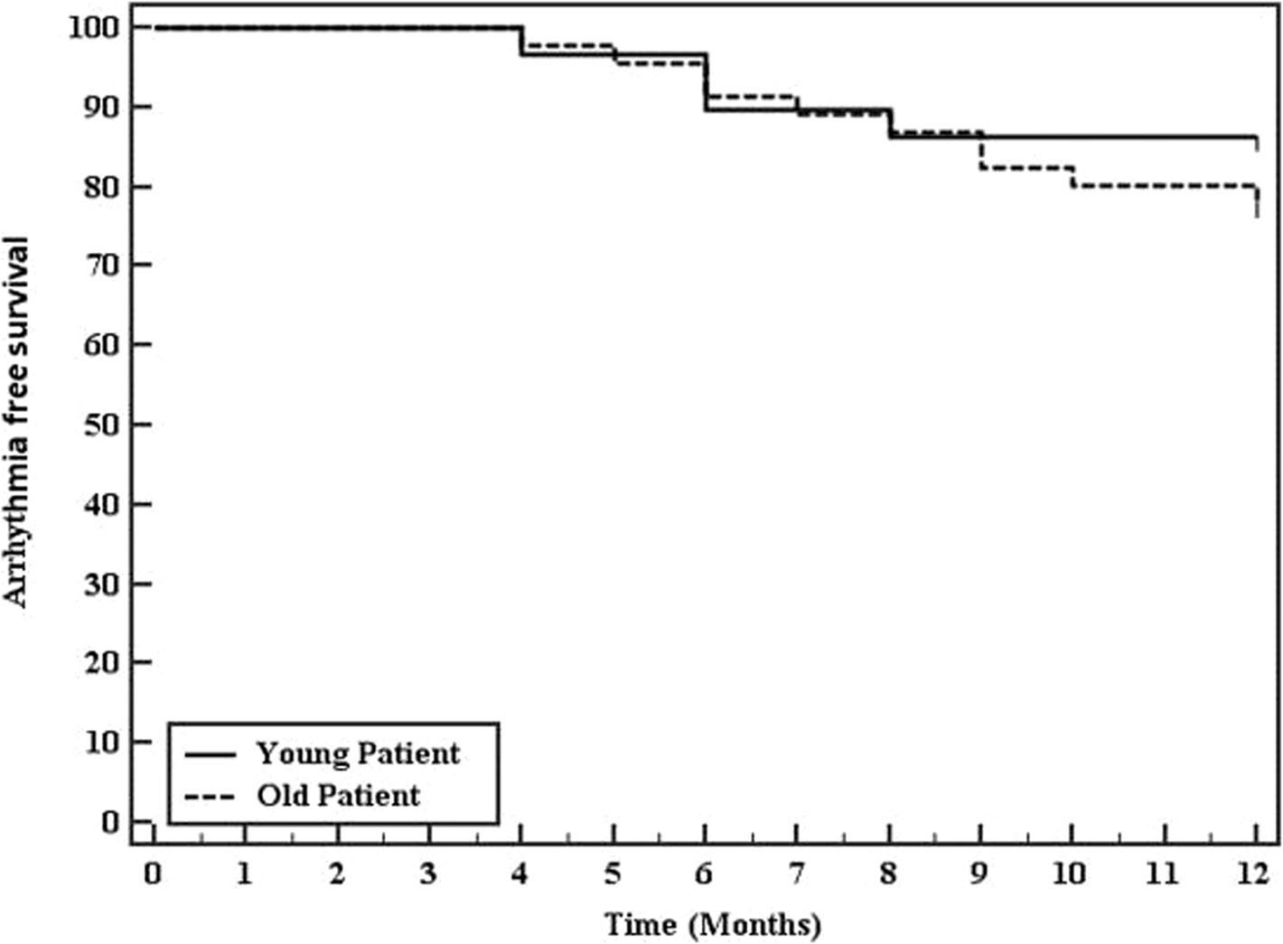
The Kaplan-Meier estimate arrhythmia-free survival and 95% confidence intervals after RF ablation of PAF in the young group (continuous line) and old group (dashed line) over 1 year. Numbers indicate remaining patients without AF recurrence at each time point

In the young population group, four of five patients (80%) who had AF recurrence responded to pharmacological treatment in the form of AADs while redo AF ablation was done in one patient (20%). In the old population group, 6 of 10 patients (60 %) responded to AADs while redo AF ablation was done in four patients (40%) (Fig. 3)

## Discussion

In the current study, we investigated the procedural characteristics and short-term clinical outcomes of RF ablation of paroxysmal AF in very young adults (< 35 years) compared to older adults (≥ 35 years). Our series showed a high 1-year arrhythmia-free survival after a single AF ablation procedure, and it was quite comparable between both younger and older study groups (83.3% vs 78.3%, respectively).

All our patients had paroxysmal AF based on clinical definitions, despite this, our mapping protocol included voltage mapping to identify the presence or absence of low voltage areas.

Several reports have demonstrated LVAs irrespective of the type of AF, and it correlated well with AF recurrence after PVI if not targeted by ablation [10, 11]. Left atrial (LA) structural alterations, especially fibrosis, are major determinants of AF pathogenesis and predict procedural failure after ablation [11–13]. Low-voltage areas (LVAs) detected by LA EA mapping correlate well with structural alterations on delayed-enhancement magnetic resonance imaging (DE MRI) and may thus serve as a suitable target for adjunctive substrate modification [14]. That is why our ablation strategy in the current study focused on the findings of voltage maps identifying LVAs as a second target for ablation after achieving complete PVI aiming at improving ablation outcomes.

### Clinical outcomes of AF ablation in two age groups

Most clinical studies that evaluated the success rate of catheter ablation for AF reported on a population with an average age of 60 years. After 5 years of follow-up and multiple procedures, success rates (± AAD) were up to 79.5% for PAF [15]. Few studies evaluated the outcome of AF ablation in younger populations ≤ 45 years, the majority of patients in these studies aged between 35 and 45 years old [16, 17] and only one study provided data on long-term follow-up in very young patients (≤ 35 years), where AF ablation was performed on a mixed patient cohort suffering from PAF or persistent AF (62% PAF) [5].

They reported a single procedural 1-year/5-year arrhythmia-free survival of 66%/44%, respectively, and a 5-year clinical success of 84% (± AAD) and 72% (off AAD) after multiple procedures. Their lower single procedural 1-year arrhythmia-free survival compared to ours (66% vs 83.3%) could be explained by their mixed study population including patients with persistent AF, different study design, and ablation protocol.

Our study is the first study to compare ablation outcomes in very young population to those of the older population in patients with PAF. A significantly higher rate of obesity and higher CHA2DS2-Vasc score were seen in the older population, which was somewhat expected, but these differences did not affect ablation outcomes or success rates.

LA scars or LVAs on voltage maps denoting LA structural alterations and fibrosis were only seen in the older population (21.7%) compared to the very young population who all showed normal voltage maps. More interestingly, the older population showed larger LA volumes compared to the very young population (*P* = 0.001). And since the AF duration was quite comparable between the two studied populations, so it can be suggested that age might have a causative relation to the structural alterations and cellular changes that end up in LA fibrosis in PAF, this hypothesis needs to be studied further. The larger LA volumes in the older population may be a cause or a result to LA scarring and creation of LVAs.

Procedural times (159.9 ± 33.1 vs 170.9 ± 30.1 min, *P* = 0.15, respectively) and fluoroscopy times (49.5 ± 28.7 vs 49.8 ± 30.6 min, *P* = 0.98, respectively) were quite comparable between younger population and older population despite the fact that ablation procedures were more complex involving substrate modification due to LA scarring in some patients in the older group. This could be explained that substrate modification involved only 21.7% of older population patients.

Our study demonstrated that RF ablation of PAF in very young patients is associated with favorable outcomes, more simple procedures confined to only PVI and a higher 1-year arrhythmia-free survival after a single procedure but is quite comparable to arrhythmias-free survival rates in older populations. This was in line with the findings of Saguner et al. [5] who reported a success rate at last follow-up in the very young population of 76% for PAF.

In our study, the need for redo procedures was quite low in the very young population when compared to the older population.

## Conclusions

The present study demonstrates that catheter ablation of AF in very young adults is associated with higher 1-year success rates but comparable to success rates in older populations. The very young patients tended to have lower rates of redo procedures in comparison to the older population.

### Study limitations

Limitations of the current study are that it comes from a single medical center with a relatively small number of patients, which limits the generalization of the results. Because of AF regression and symptom improvement with re-initiation of AADs in case of AF recurrence, there may be a potential for under-recognition of asymptomatic AF episodes. This study is limited also by its relatively short-term follow-up for just 1 year.

## Data Availability

They are available from the corresponding author on reasonable request.

## Abbreviations

3D EA mapping: Three-dimensional electro-anatomical mapping
6MWD: 6 min walking distance test
AAD: Antiarrhythmic drugs
ACT: Activated clotting time
AF: Atrial fibrillation
AP: Accessory pathway
AVNRT: Atrio-ventricular nodal reentrant tachycardia
AVRT: Atrio-ventricular reciprocating tachycardia
BMI: Body mass index
DCM: Dilated cardiomyopathy
ECG: Cardiac electrocardiogram
EF: Ejection fraction
EGM: Intracardiac electrocardiogram
HCM: Hypertrophic cardiomyopathy
IHD: Ischemic heart disease
INR: International normalized ratio
LA: Left atrium
LIPV: Left inferior pulmonary vein
LSPV: Left superior pulmonary vein
LVA: Low-voltage area
MA: Mitral annulus
PAF: Paroxysmal atrial fibrillation
PV: Pulmonary veins
PVPs: Pulmonary vein potentials
RFCA: Radiofrequency catheter ablation
RIPV: Right inferior pulmonary vein
RSPV: Right superior pulmonary vein
SHD: Structural heart disease
VHD: Valvular heart disease,
